# Making the Most of Major Histocompatibility Complex Molecule Multimers: Applications in Type 1 Diabetes

**DOI:** 10.1155/2012/380289

**Published:** 2012-05-28

**Authors:** Greg S. Gojanovich, Paul R. Hess

**Affiliations:** Department of Clinical Sciences and Immunology Program, College of Veterinary Medicine, North Carolina State University, 1060 William Moore Drive, Raleigh, NC 27607, USA

## Abstract

Classical major histocompatibility complex (MHC) class I and II molecules present peptides to cognate T-cell receptors on the surface of T lymphocytes. The specificity with which T cells recognize peptide-MHC (pMHC) complexes has allowed for the utilization of recombinant, multimeric pMHC ligands for the study of minute antigen-specific T-cell populations. In type 1 diabetes (T1D), CD8+ cytotoxic T lymphocytes, in conjunction with CD4+ T helper cells, destroy the insulin-producing **β** cells within the pancreatic islets of Langerhans. Due to the importance of T cells in the progression of T1D, the ability to monitor and therapeutically target diabetogenic clonotypes of T cells provides a critical tool that could result in the amelioration of the disease. By administering pMHC multimers coupled to fluorophores, nanoparticles, or toxic moieties, researchers have demonstrated the ability to enumerate, track, and delete diabetogenic T-cell clonotypes that are, at least in part, responsible for insulitis; some studies even delay or prevent diabetes onset in the murine model of T1D. This paper will provide a brief overview of pMHC multimer usage in defining the role T-cell subsets play in T1D etiology and the therapeutic potential of pMHC for antigen-specific identification and modulation of diabetogenic T cells.

## 1. Introduction

Due to antigen-specific assaults on the insulin producing beta (*β*) cells of the pancreas by diabetogenic T cells, insulin synthesis and secretion is lost, resulting in dysregulation of blood glucose homeostasis and aberrant lipid and protein metabolism in type 1 diabetic (T1D) patients. T1D is one of the most prevalent chronic autoimmune diseases in the United States and affects ~36 million individuals worldwide [1, 2]. The incidence of T1D is predicted to increase worldwide by 3% annually [3]. With administration of exogenous sources of insulin via daily injections, continual pump therapy, or islet replacement transplants, diabetics can currently enjoy near normal life spans and reduced incidence of heart disease, kidney failure, blindness, and neuropathy [4]. However, none of the currently available therapies can adequately quell the long-term risks of hypoglycemia or microvascular damage and the financial expense of treatment [1, 4]. In addition, the total direct and indirect costs associated with diabetes in 2007 were $174 billion dollars in the United States [1]. Therefore, new therapies are needed to reduce the burden of this chronic disease on both the individual and society.

The aforementioned T1D therapies are directed only at the consequences of the disease rather than the causative agent(s). While the ultimate cause of T1D is not currently defined and is likely multifactorial [5], T cells specific for islet antigens are the proximate cause of *β* cell destruction. Diabetogenic T cells that have been inappropriately activated and allowed to escape central and peripheral tolerance mechanisms (possibly due to assistance from aberrant innate cells [6]) have been implicated in *β* cell damage in both the nonobese diabetic (NOD) mouse and the human form of disease. In peripheral lymphoid organs and in circulation, CD4+ T cells and CD8+ T cells interact with peptide-major histocompatibility complex class II or class I (pMHCII or pMHCI) molecules, respectively. MHCII molecules are externally displayed on the cell surface of professional antigen presenting cells (APCs) and contain peptide fragments primarily derived from extracellular proteins, while classical MHCI molecules are found on the surface of all nucleated cells and present peptides of cytosolic origin. In T1D, diabetogenic CD4+ T cells (T_H_) are responsible for providing the cytokine microenvironment in which islet-specific CD8+ T cells (CTL) destroy *β* cells. Furthermore, islet-specific T_H_ activity may also be cytotoxic to islets at later disease stages, and CTL may not need prior costimulation before killing *β* cells [7–11]. Thus, both T_H_ and CTL are contributors to diabetes pathogenesis and are suitable targets for strategies aimed at preventing T1D. However, it is currently unknown whether T1D preventative strategies will be more effective when targeting CTL or T_H_, and future therapies will likely target both subsets of autoreactive T cells.

The ability to modulate the behavior of T cells is therefore an approach for novel treatments in T1D and other T-cell-mediated diseases (e.g., multiple sclerosis, transplant rejection). Most T-cell-targeting strategies have utilized non-specific or blanket therapies, such as Thymoglobulin or anti-CD3 monoclonal antibodies, that target all T cells. Therefore, these treatments may also prevent protective immunity by the adaptive immune system, resulting in susceptibility to new infections, viral reactivation, or increased risk of cancer [12, 13]. Thus, researchers have pursued various means of exclusively modulating the autoreactive T cells with antigen, which can be delivered covalently linked to tolerizing cells, within viral vectors, or as whole proteins or soluble peptide fragments [14, 15]. Many studies have addressed the identity of cognate antigens for diabetogenic T cells in mouse and human T1D [16], but the relative importance of each peptide is still unknown for human disease [17]. While the repertoire of diabetogenic T-cell epitopes has been investigated extensively, the molecular form of antigen (e.g., whole protein versus peptide fragments), the route of delivery, or modifications such as carrier proteins or adjuvant combinations still need to be more clearly defined in order to produce efficient therapeutic strategies [15, 18].

One major obstacle to the use of soluble peptides to target antigen-specific T cells is the relatively short half-life of these molecules once administered. To remedy this, researchers have designed vehicles that mimic the natural setting in which a peptide is presented to T cells; that is, peptides are combined *in vitro* with purified or recombinant MHC molecules to create soluble, stable complexes [19, 20]. These peptide-MHC (pMHC) complexes are then able to bind to cognate TCR on antigen-specific T cells. The binding affinity of the TCR to a single pMHC molecule is in the weak micromolar range but can be increased by producing pMHC multimers. The multimeric form allows for numerous pMHC-TCR interactions to occur simultaneously on the surface of a cognate T cell to synergistically increase the binding avidity into the nanomolar range [21]. This advancement has allowed for identification and enumeration of T-cell populations that are found at frequencies of less than 10^−5^. The most common form of pMHC multimer currently used is the tetramer, which is produced by linking four biotinylated pMHC complexes via streptavidin. In most forms, pMHC multimers can be labeled for use in flow cytometry, magnetic resonance imaging, or modulation of antigen-specific T-cell activity. This paper will cover how pMHC multimers have been used to unravel T1D pathogenesis and how these agents may be applied to treat the disease.

## 2. Peptide-Major Histocompatibility Complex Multimers in T1D

Our understanding of islet-specific T cells in the pathogenesis of T1D has been greatly enhanced following the elucidation of major *β* cell autoantigens and the creation of pMHC multimers specific for diabetogenic T-cell subsets. In fact, the use of these tools may allow for more accurate prognoses than the currently used predictions based on islet autoantibody titers. For example, researchers can collect blood samples from at-risk individuals and evaluate T_H_ and CTL specific for islet antigens using fluorochrome-labeled pMHC tetramers. While earlier studies of autoreactive T cells in peripheral circulation required *ex vivo* peptide stimulation in order to detect antigen-specific T_H_ cells via multimer-labeling [22, 23], advances in pMHCII technology have allowed for direct detection of diabetogenic T cells in lymph nodes of NOD mice [24], indicating that pMHC technology may eventually allow for enumeration of autoreactive T_H_ clonotypes in peripheral circulation. Alternatively, the use of ELISPOT techniques, while still requiring a stimulatory incubation period, also allows for enumeration of islet-reactive T cells in diabetics [25] though the differences between pMHC multimer and ELISPOT assays in detecting islet-specific T cells that physiologically function as diabetogenic clonotypes remain to be clarified [26]. Utilizing pMHCI multimers to label the blood cells of young NOD mice, elevated numbers of CTL specific for the NRP mimotope, a synthetic analog of the islet antigen IGRP_206–214_, were found to strongly correlate with the degree of insulitis [27]. Importantly, this correlation predicted the onset of overt diabetes prior to detection of hyperglycemia. In a similar fashion, investigators detected pMHCI-specific autoreactive CTL in the blood of recent-onset diabetics that were not present in control subjects [28]. The presence of islet-reactive CTL in peripheral blood also correlated with impending insulin dependence in islet transplant recipients. Thus, pMHC multimers offer clinically relevant tools for T1D that may surpass current diagnostic protocols.

In addition to the value of pMHC multimers as diagnostic tools for blood specimens, this technology has led to demonstrations of the presence and expansion of diabetogenic T cells within secondary and tertiary immune compartments. Liu et al. were the first to describe the presence of glutamic acid decarboxylase (GAD)65-reactive T_H_ cells in the lymphoid tissues of NOD mice by utilizing tetramers composed of murine MHCII molecules that were complexed with GAD65-derived peptides [22]. Similar protocols and results were described in recent-onset human T1D patients [23]. Wong et al. utilized pMHCI tetramers loaded with an insulin-derived peptide epitope (determined via cDNA screening libraries) to demonstrate CTL infiltration into islets of young NOD mice, finding that insulin B_15–23_-reactive CTL comprised a large proportion of islet infiltrating T cells and that this population decreased over time [29]. Studies by Amrani et al. indicated that NRP-A7-specific CTLs predominate as animals age [30] and that this phenomenon occurs due to a process deemed “avidity maturation,” in which higher-avidity, more aggressive IGRP-reactive CTL clonotypes expand within the islets over time, likely due to a lack of tolerance mechanisms that restrict the clones as disease progresses [31].

Such insights could indicate that there is a primary clonotype responsible for T1D initiation—but which one? Krishnamurthy et al. demonstrated that NOD mice tolerized to proinsulin do not develop IGRP-specific CTL infiltration into islets and, importantly, do not become diabetic, while those tolerized to IGRP still developed proinsulin-reactive CTL, resulting in T1D [32]. These results support the idea of a driver clonotype (proinsulin-reactive) and the recruitment of secondary effectors (IGRP-reactive) through the phenomenon of “epitope spreading.” Thus, it may be difficult to determine which specificity to target with antigen-specific therapy, particularly in light of the variability of diabetogenic CTL populations found in both mice and humans. For example, Lieberman et al. compared CTL specificities in age-matched NOD mouse islets at different time points and found different antigen-specific clonotypes predominating in each animal [33]. Similarly, research involving recent-onset human diabetics also found no immunodominant CTL reactivity across individuals [34]. Again, such variability could arise from epitope spreading; alternatively, these data could signify that there is no universal, underlying epitope responsible for T1D induction in either species. Even this worst case scenario—where private T-cell specificities initiate diabetogenesis—does not automatically doom antigen-specific-based treatment as a practical therapy for T1D, because of immunodominance, which likely restricts these specificities to one or a few epitopes per MHC haplotype. Thus, a limited number of antigen-specific agents presumably would cover a large percentage of the diabetic patient population. This restriction is reinforced by the relative dominance of some class I (HLA-A*0201, B*3906, or C*0501) and class II (DPA1*0103-DPB1*0202 or DRB1*0405-DQA1*0301-DQB1*0302) alleles in T1D [35].

Whether there is a primum movens diabetogenic T-cell specificity is debatable; however, T-cell trafficking to the islets clearly occurs prior to overt diabetes. Tetramers labeled with magnetic nanoparticles have allowed real-time magnetic resonance imaging of NRP-specific CTL infiltrating NOD islets [36, 37]. Such a noninvasive procedure could be invaluable for the detection of insulitis in at-risk patients, further supporting the use of multimerized pMHC as diagnostic measurements of T1D onset.

In addition to the use of pMHC multimers described previously, these constructs can also be employed to manipulate both T_H_ and CTL subsets *in vivo.* The strategy of pMHC therapy in T1D is based on TCR-pMHC binding in the absence of costimulatory or cytokine signals, thereby inducing anergic or regulatory T-cell phenotypes. Numerous studies have described the possible outcomes, such as full priming, partial activation, unresponsiveness, or induction of apoptosis, following administration of pMHCI to CTL *in vitro *and *in vivo* [10, 38–40]. Similar results have been detected in human T_H_ treated with pMHCII multimers [41, 42]. Such varying effects of pMHC administration upon modulation of diabetogenic T cells will be highlighted later, but are likely due to pMHC multimer avidity, functional plasticity of the clonotype targeted, and the molecule conjugated to the pMHC multimer. Specifically, shortened linkage length between the pMHCI monomers in multimeric complex correlated with induction of CTL apoptosis [38], and the numbers, or valence, of pMHCII monomers in complex was determined to affect T_H_ fate [43]. Alternatively, if the goal is to delete specific T-cell subsets, pMHC multimers may be used to deliver toxic moieties to cognate T cells [44–47].

Advantages of pMHC multimer therapies over “blanket” or mAb-based treatments include the capacity to rapidly create “designer” pMHC multimers that directly target diabetogenic T cells. Specifically, pMHCI folding protocols can incorporate photocleavable peptides into the MHCI binding groove that can be rapidly replaced by peptides of interest following exposure to UV light, while still maintaining the MHCI complex [48]. This technique was recently applied to human T1D patients and led to the discovery of new preproinsulin epitopes against which T-cell frequencies were found to correlate with islet transplant rejection outcomes [49]. Unlike in toxic mAb therapies, diabetogenic T cells cannot be selected to escape targeting by toxic multimers because the pMHC is the ligand for the TCR, which is necessary for recognizing and killing *β* cells. However, certain issues will need to be addressed before pMHC become clinically acceptable treatments for T-cell-mediated diseases. Firstly, multimer therapy in its current form may be immunogenic, resulting in antimultimer antibodies production following repeated administration. Secondly, some studies have indicated that pMHC therapy may actually activate T cells and exacerbate disease due to the transfer of peptides from multimers to native MHC on host tissues [50]. Yet, the first concern could be addressed by altering the structural design of the therapeutic pMHC to reduce immunogenicity, while covalent linkage of peptides to the multimer could obviate the second issue [40]. Lastly, pMHC administration may induce refractory periods during which T cells are transiently resistant to binding pMHC complexes [51]. This issue could be addressed by determining the optimal dosing interval in order to avoid delivery during such periods. While unanticipated technical problems are likely to arise, the ability to manipulate T cells in an antigen-specific manner warrants further investigation into pMHC therapy for T1D.  Table 1 summarizes the designs and outcomes for pMHC-based experiments targeting autoreactive CTL and T_H_ cells in diabetes models.

### 2.1. CD4+ T-Cell-Directed Multimer Usage: pMHCII

As mentioned previously, T_H_ cells, along with innate immune cells, are responsible for creating an inflammatory microenvironment within islets undergoing insulitis. This cytokine milieu usually includes IL-2, IFN-*γ*, TNF-*α*, and IL-12, which promote cellular immune responses and inflammation and may inhibit IL-4 and IL-10 production. One goal of pMHCII multimer-based immunomodulation therapy is to shift the T_H_-cell-mediated inflammatory state to one of a regulatory phenotype [57]. In a double transgenic T1D mouse model expressing influenza hemagglutinin (HA) under the rat insulin promoter, and in which CD4+ T cells are specific for the HA antigen, Casares et al. demonstrated the ability of pMHCII dimers to prevent T1D onset in prediabetic mice and to restore euglycemia in newly diabetic mice [54]. While untreated mice usually become diabetic within 10 weeks of age, frequent dimer treatment induced T regulatory cells that produced IL-10 within the pancreatic islets and also induced anergy of antigen-specific T_H_ cells in the spleen, resulting in extended durations of normoglycemia. In an adoptive transfer model of T1D, BDC2.5 T_H_ cells (specific for chromogranin A_29–24_ [58]) are capable of inducing T1D onset in T-cell deficient NOD recipients [55]. However, when recipients were treated with pMHCII dimers that target the BDC2.5 T cells, T1D was prevented due to the conversion of BDC2.5 T_H_ into regulatory-type cytokine producers. Specifically, this effect was attributable to IL-10, as shown by diabetes progression in mice treated with antibodies that block IL-10 receptors. However, the utility of pMHCII dimer treatment was not translatable to wild-type (WT) NOD mice that contain T_H-_cells specific for numerous other islet antigens. Yet, more recent work with prediabetic WT NOD mice has confirmed previous studies in that pMHCII dimers do indeed induce T-cell phenotype conversion and can effectively block progression to overt diabetes if T_H_ specific for GAD65-derived epitopes are targeted [56], possibly indicating that responses to this antigen occur prior to those directed at the chromogranin A epitope.

A second aim of pMHCII multimer therapy is the antigen-specific deletion of autoreactive T_H_ cells. Such therapies attempt to prevent T1D pathogenesis by eliminating T_H_ populations, thus preventing the development of an inflammatory microenvironment and/or the full activation of islet-specific CTL. The first efforts at clonal deletion of antigen-specific T_H_ cells utilized toxic moieties, doxorubicin or mycophenolate, conjugated to pMHCII multimers [44, 45]. However, studies that expand on these initial findings in T1D have not been reported. In another work, Preda-Pais et al. utilized the double transgenic HA mouse model described previously and determined the effect of increasing the numbers of pMHCII monomers in multimeric complexes (thus increasing avidity for cognate TCRs) [43]. Clonal deletion of antigen-specific T_H_ cells was achieved using octameric pMHCII complexes, which resulted in prolonged protection from T1D, as compared to treatment with the pMHCII dimer previously used by the same group. Further studies regarding the efficacy of pMHCII multimers in preventing T1D via clonal T_H_ cell ablation in WT NOD mice have not yet been described. Thus, pMHCII-based therapies show promise in their capacity to delay or prevent overt T1D in susceptible mice via induction of anergic or regulatory phenotypes and by deletion of diabetogenic T_H_ cells.

### 2.2. CD8+ T-Cell-Directed Multimer Usage: pMHCI

Immunomodulation of CTL activity via pMHCI multimers was first described by Dal Porto et al., who created dimeric MHCI-Ig fusion proteins that were capable of inhibiting alloreactive CTL *in vitro* [21]. Subsequently, other studies confirmed that pMHCI multimers can inhibit CTL activity *in vivo* [59, 60]. The mechanisms by which pMHCI multimers modulate aggressive activity of CTL were addressed in a murine model of tissue rejection by Maile et al. [39]. This study utilized the male HY alloantigen to determine the capacity of pMHCI multimer treatment to prevent tissue rejection *in vivo*. Administration of pMHCI to naïve female recipients of male skin grafts induced a CD8^lo^ phenotype in allospecific CTL, which corresponded to antigen hyporesponsiveness and the production of TGF*β*. Thus, pMHCI tetramer treatment induced immunosuppressive CTL in female mice that prevented other alloreactive CTL from rejecting male tissue grafts. Results of these studies indicate that immunomodulation of diabetogenic CTL clonotypes might also be feasible. However, when pMHCI multimers targeting IGRP-reactive CTL for immunomodulation were administered to NOD mice, diabetes was not prevented (unpublished observations).

More recently, promising results for pMHCI multimers have been obtained in both WT and humanized (transgenic HLA-A*0201 expression) NOD mice [52]. Tsai et al. utilized iron-oxide nanoparticles coated with antigenic islet epitope-displaying MHCI molecules to determine the effects on diabetogenic CTL. Surprisingly, pMHCI-nanoparticle treatment boosted numbers of the antigen-specific CTL clonotype, but prevented or reversed diabetes in pre- and newly-diabetic NOD mice. This finding was attributed to the expansion of low avidity, autoregulatory (or suppressor) CTL that are capable of suppressing autoimmunity via IFN*γ*-, indoleamine-2, 3-dioxygenase-, and perforin-dependent mechanisms in a non-antigen-specific manner. Thus, pMHCI-based therapies may prevent and even counteract insulitis in T1D-susceptible individuals via enhancement of autoregulatory mechanisms.

**Figure 1:**
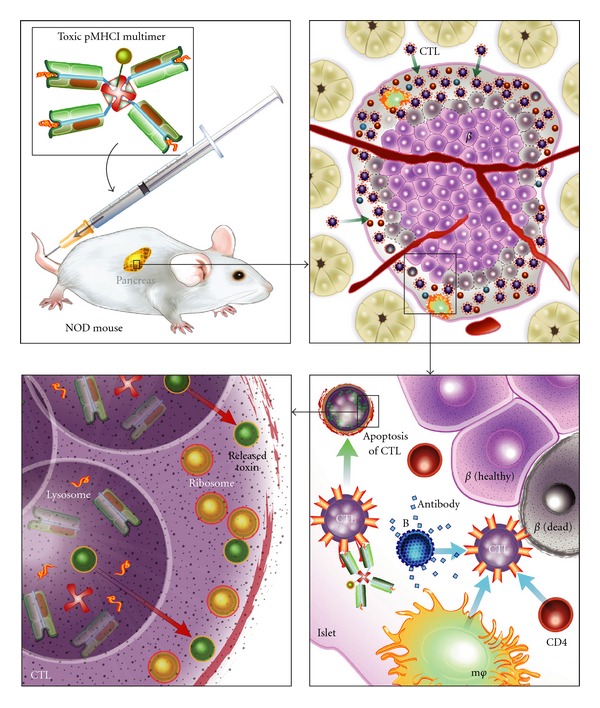
Schematic representation of the effects of toxic pMHCI tetramer administration to NOD mice. Clockwise from upper left: toxic moieties, such as saporin, can be linked to pMHCI multimers that are specific for diabetogenic CTL before being delivered to prediabetic NOD mice. Toxic pMHCI tetramers can access inflamed islets where cognate CTLs are present. Insulitis involves numerous autoreactive immune cell subsets that act in concert to potentiate diabetogenic CTL and destroy *β* cells. However, pMHCI multimers can deliver saporin to diabetogenic CTL clonotypes and induce apoptosis in an antigen-specific manner. Cognate TCR interacting with saporin-conjugated pMHCI results in uptake of the tetramer into the lysosome of the CTL. In the lysosome, the toxic pMHCI tetramer is disassociated, allowing the freed saporin molecules to escape these organelles. Saporin is a potent ribosome-inactivating toxin that can perform multiple rounds of ribosome disabling, ultimately leading to apoptosis of the CTL and therein preventing further *β* cell damage by the targeted diabetogenic clonotype.

While pMHCI-mediated expansion of islet-specific suppressor CTL appears to hold great promise for recent-onset T1D treatment, it is plausible that such therapies would be ineffective for modulating all islet-specific CTL, such as memory-like CTL clonotypes found in long-term diabetics. Therefore, the use of pMHCI multimers to specifically ablate diabetogenic clonotypes may be advantageous for islet transplantation, since islet antigen-specific CTL clonotypes mediate recurrence of T1D [61–65]. The first description of a toxic pMHCI multimer was published by Yuan et al. [46] and utilized tetramers conjugated to a radionuclide, which eliminated antigen-specific CTL *in vitro*. Using saporin as a toxic agent, we demonstrated the ablation of transgenic CD8+ T cells in an antigen-specific manner *in vitro *and* in vivo* [66]. Our group then demonstrated that toxic pMHCI administration killed IGRP-reactive CTL transferred into T-cell deficient NOD.*scid* recipients [53]. Toxic tetramer treatment also resulted in the rapid reduction of cognate CTL (>75% eliminated 72 hours post treatment) from the spleens of lymphoreplete NOD recipients. Furthermore, toxic tetramer-treated WT NOD mice showed delayed onset of T1D (3 of 10 treated animals were euglycemic at study completion, compared to none among the PBS-injected mice), and lasting depletion (for 44 weeks after treatment) of cognate CTL from the islets. Lastly, treatment was initiated at 8 weeks of age, a time point when the autoimmune response is well underway in NOD mice, indicating that this strategy may be applicable to the prevention of disease progression.  Figure 1 depicts parenteral administration of pMHCI tetramers, conjugated to the ribosome-inactivating molecule saporin, which can diffuse into pancreatic tissues and interact with diabetogenic CTL at the site of inflammation. Binding of toxin-linked pMHCI to the cognate TCR results in the uptake of the multimer into the lysosomes of the T cell, where the toxin is then cleaved from the tetramer. Released saporin escapes into the cytosol, inactivating ribosomes and thereby killing the CTL, which prevents *β* cell destruction. Thus, immunomodulation and ablation strategies utilizing pMHCI multimers may be suitable for preventing progression or recurrence of T1D.

## 3. Conclusions

In summary, the goals of pMHC multimer usage in T1D are to create a better prognostic indicator for diabetes onset or impending islet transplant rejection; to halt the destructive activity of diabetogenic T-cell clones via anergy induction, phenotype conversion, or clonal deletion; and ultimately, to restore the balance of islet-specific effector and suppressor T cells in at-risk individuals. Future studies should address the following issues with pMHC multimer administration: determining optimal time point of therapy application (is there an ideal time to deplete antigen-specific CTL?) utilizing numerous pMHCI multimers of different specificities simultaneously (can we modulate multiple CTL populations?) combining therapies that target CD4+ and CD8+ T cells (would targeting T_H_ and CTL have a synergistic effect?); combining therapies that target other cell types (will B cell-depleting antibodies prevent epitope spreading?) and producing next generation recombinant pMHC molecules (can we more effectively target islet-reactive T cells?). Therefore, we believe that this technology will continue to evolve and perhaps be used in conjunction with existing therapies to treat or prevent T cell-mediated autoimmune disorders such as T1D.

## Figures and Tables

**Table 1:** Summary of studies investigating therapeutic effects of pMHC multimer treatment on T1D in mouse models.

MHC restriction	Mouse model	Antigen*	Multimer form	Modification	T1D status at administration	Therapeutic effect	Mechanism of modulation	Reference, year
Class I	NOD	NRP; mIGRP; DMK	Nanoparticle	None	Prediabetic	Protected	Potentiated low avidity, regulatory CD8+ T cells	Tsai et al., 2010 [52]
	—	—	—	—	Newly diabetic	Reversed		
	Humanized NOD	hIGRP; InsB	—	—	—	Reversed		
	NOD	NRP; mIGRP	Tetramer	Toxin	Pre-diabetic	Delayed onset	Deleted cognate CD8+ T cells	Vincent et al., 2010 [53]
	—	DMK; InsB	—	—	—	No protection		
Class II	TCR x beta cell antigen double transgenic cross	HA	Dimer	None	Pre-diabetic	Protected during treatment	Induced anergy and IL-10- secreting Tregs	Casares et al., 2002 [54]
	—	—	—	—	Newly diabetic	Reversed		
	—	—	—	—	Late diabetic	No reversal		
	Transfer of transgenic CD4+ T cells	ChgA	Dimer	None	Pre-diabetic	Protected during treatment	Induced anergy and IL-10- secreting cells	Masteller et al., 2003 [55]
	Transfer of NOD splenocytes; NOD	—	—	—	Pre-diabetic; diabetic	No protection; No reversal		
	TCR x beta cell antigen double transgenic cross	HA	Octamer	None	Pre-diabetic	Delayed onset	Caused activation-induced cell death	Preda-Pais et al., 2005 [43]
	—	—	—	—	Newly diabetic	Reversed		
	NOD	GAD65	Dimer	None	Late pre-diabetic	Protected	Induced GAD-specific, IL-10-secreting Tregs	Li et al., 2009 [56]

*mIGRP, murine islet-specific glucose-6-phosphatase catalytic subunit-related protein; DMK: dystrophia myotonica kinase; hIGRP: human IGRP; InsB: insulin B chain; HA: influenza hemagglutinin; ChgA: chromogranin A; GAD65: glutamic acid decarboxylase 65. ^—^Indicates content is identical to entry directly above.
